# Validation of Parmigiano Reggiano Cheese Aroma Authenticity, Categorized through the Use of an Array of Semiconductors Nanowire Device (S3)

**DOI:** 10.3390/ma9020081

**Published:** 2016-01-28

**Authors:** Veronica Sberveglieri

**Affiliations:** CNR-INO Sensor Lab, Via Branze, 45, Brescia 25123, Italy; veronica.sberveglieri@ino.it; Tel.: +39-3204-377-973

**Keywords:** nanowire gas sensors, Parmigiano Reggiano, gas sensor device, GC-MS, aroma characterization

## Abstract

Parmigiano Reggiano (PR) cheese is one of the most important Italian Protected Designation of Origin (PDO) cheeses and it is exported worldwide. As a PDO, the product is supposed to have distinctive sensory characteristics. In this work we present the use of the Small Gas Sensor System (S3) device for the identification of specific PR markers, as compared to classical chemical techniques, such as Gas chromatography–mass spectrometry solid-phase microextraction (SPME-GC-MS). Markers are used to determine the percent of grated pulp and rind commercially utilized. The S3 device is equipped with an array of six metal oxide semiconductor (MOX) gas sensors, three of them with a nanowire (NW) morphology and the other three in the form of thin films. PDO can cover grated PR cheese as well, but only if made with whole cheese. Grated PR cheese must be characterized by the absence of additives and no more than 18% crust. The achieved results strongly encourage the use of S3 for a rapid identification of the percentage of grated PR.

## 1. Introduction

Parmigiano Reggiano (PR) cheese is one of the oldest traditional cheeses produced in Europe (XII century) and it is still one of the most esteemed Protected Designation of Origin (PDO) Italian cheeses [1]. PR Cheese originates in Northern Italy, in the countryside area that touches five different cities (Parma, Reggio Emilia, Modena, and portions of the provinces of Mantua and Bologna). PR is a hard curd cheese obtained through a peculiar technology based on the use of unheated raw cow’s milk.

For a cheese to gain the Protected Designation of Origin (PDO) title, the bond with its own traditional characteristics is dramatically important because, as stated in the Council Regulation European Union (EC) No. 510, 2006 on the protection of geographical indications and designations of origin for agricultural products and foodstuffs, the link between “authentic and unvarying local methods” and “tradition” is essential. This indication implies that PDO products also have distinctive sensory characteristics, which are strongly connected to the traditional production methods, thus guaranteeing the consumer a sensory quality that lasts over time.

The PR cheese Consortium (CFPR), located in the PR production area (Italy), is the institutional system that covers all cheese dairy factories and controls cheese production in terms of cow feeding, cheese manufacturing, and ripening processes.

CFPR production guidelines are based on the secular experience, the regional habits and practices, and the technological innovations that could leave the uniqueness of the product unchanged (EC, 510/2006) [2]. Research on aroma chemistry have attracted a lot of attention, as well.

The grating operation must take place in the defined geographical area of cheese production and packaging must start immediately, without any processing or addition of substances that could change its preservation and its original organoleptic characteristics.

Grated cheese has to be characterized by the absence of additives, no more than 18% crust in its moisture, a non-crumbly aspect and homogeneous particles with less than 0.5 mm diameter, not exceeding 25% (Decreto del Presidente del Consiglio dei ministri della Repubblica Italiana, D.P.C.M., 4/11/1991). Therefore, it is essential to find a method to determine the rind and pulp percentage of grated PR cheese, based not only on the classical expensive and slow chemical techniques. Classical techniques like High Performance Liquid Chromatography (HPLC), GC-MS, or microbiological analysis need a few hours to achieve results.

The possibility of detecting the addition of extra rind to grated cheese (addition made with a large range of different seasoned cheeses) and the ripening time of PR cheese has been studied by means of destructive chemical methods.

The fight against counterfeiting is a priority for PDO of protection. The globalization of markets has been providing both new opportunities and risks: the possibility to package the product worldwide makes carrying out document checks increasingly difficult and this is why the Consortium is working to change the rules of production of Parmigiano Reggiano requiring conditioning in the area, and developing sophisticated solutions for product traceability.

The aim of this work is to provide a rapid, non-destructive, and compact device able to determine the authenticity of PR and the respect of its regulations (grated percentage).

Aroma (fingerprint) is one of the most significant food parameters from a sensory point of view and it may offer information about safety and quality, sometimes being an indicator for processing mistakes, as well [3].

The use of an S3 device to determine the specific volatile organic compounds (VOCs) of grated PR is a promising approach offering, at the same time, a simpler, faster, and easier to handle (no specialized technicians are required) solution if compared to other analytical techniques (GC-MS, HPLC).

From a commercial point-of-view, low cost and reduced power consumption are fundamental features. Gas sensor arrays or S3 devices (also called an “electronic nose”) have been identified in the last few years as valuable candidates for different applications, including food quality control [4].

## 2. Results and Discussion

### 2.1. GC-MS-SPME

Through the use of GC-MS-SPME this work could identify about thirty volatile compounds responsible for the identification and classification of our grated matrix, all of these compounds widely reported in literature [5]. These compounds are fundamental to obtain a solid results structure, able to unequivocally characterize the analyzed samples.

After examining the chromatograms of individual samples and removing the insignificant compounds, we selected about 30 key compounds to be used to create the matrix. These compounds were then compared to draw some considerations (discussed below).

The different mixtures of pulp/crust compounds identified through GC-MS-SPME were then grouped into different families to check whether there were any macroscopic differences in terms of type and number [3].

The analysis identified specific markers for the crust: 2-heptanone, 2-octanone, and 2-nonanone (considering 18% crust is the limit to the maximum percentage by law, ketones are much more present in the crust than in the pulp). The specific markers identified in the pulp were: phenyethyl alcohol and 2-2'-Oxybis ethanol (alcohol) (Table 1) [1].

**Table 1:** List of the selected chemicals compounds.

# Peak	Retention Time (RT)	Compound	Crust	Pulp
1	1285	2-heptanone	x	–
2	1745	2-octanone	x	–
6	1997	5-Methyl-2-hexanol	–	x
3	2160	2-nonanone	x	–
4	3803	phenyethyl alcohol	–	x

It was also possible to identify other specific markers for the crust PR: 8-Nonen-2-one (ketone), 2-nonanol.

SPME-GC-MS analysis suggests that, in general, alcohols typically characterize the pulp, while ketones, and free fatty acids can be more frequently found in the crust.

**Figure 1:**
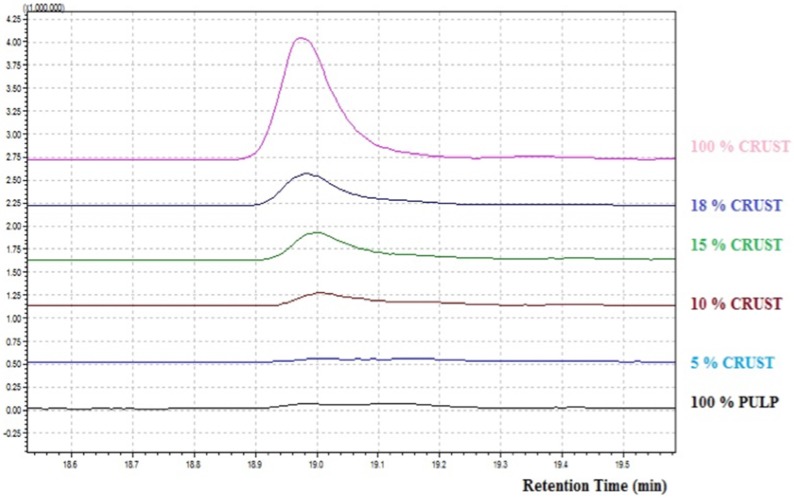
SPME-GC-MS, 5-Methyl-2-hexanol identified in 6 different percentages of grated PR.

Ccompound 5-Methyl-2-hexanol (Figure 1), identified through SPME-GC-MS is present in all analyzed samples. Its quantity decreases considerably between crust and pulp. In literature it is reported as typical VOCs present in the wood and used to ripen the cheese.

This compound is in fact completely absent in the sample made of 100% pulp (which is not in contact with seasoning tables). This compound was identified as a specific marker.

### 2.2. S3 Device

**Figure 2:**
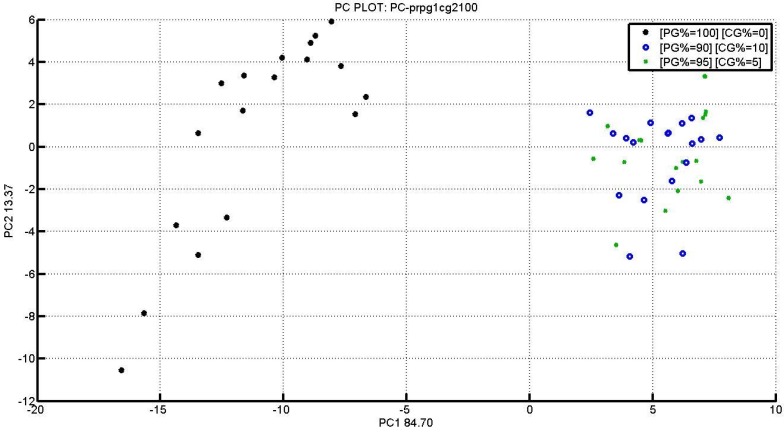
PG1 and CG2 PCA Score PLOT with different crust percentages.

Data were processed by PCA (principal component analysis) with Matlab software. Information thus obtained (Figure 2) with PC1 and PC2 were completely explained to differentiate the two present clusters [6,7,8,9,10,11,12].

The figure shows three different percentages, 100% pulp, 90%, and 95%. Therefore, the S3 device was able to differentiate samples containing crust or only pulp.

**Figure 3:**
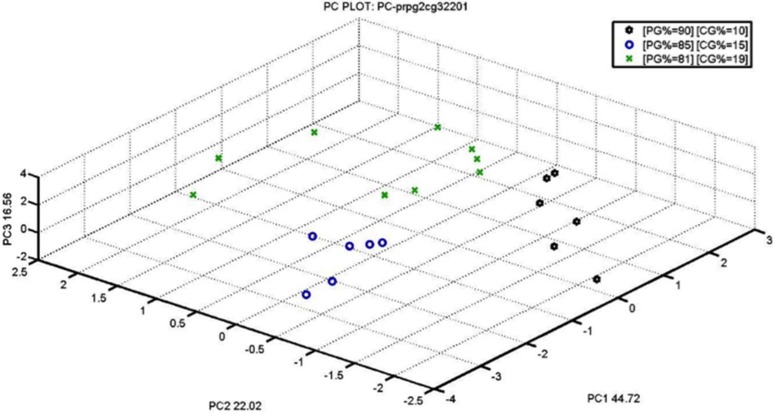
PCA Score PLOT for different crust percentage (10%, 15%, and 19%).

Figure 3 shows three different clusters formed by three different percentages of blend crust/pulp (10% black stars, 15% blue circle, and 19% green cross). PCA clearly explains the efficiency of S3 technology for differentiation.

**Figure 4:**
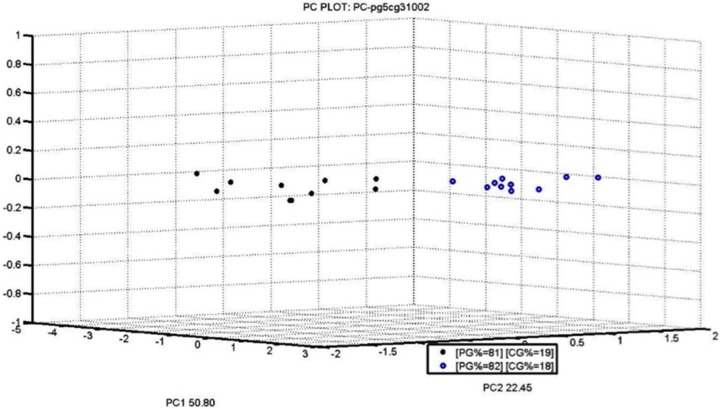
PCA Score PLOT for two different analyzed percentages (18% and 19%).

PCA obtained with our S3 (Figure 3 and Figure 4) is extremely significant because it compares two similar percentages close to the legal limit: PG5 81%–19% CG3 (not legal), PG5 82%–18% CG3 (legal limit).

The two groups of samples are very compact and clearly recognized by the nanowire sensors fraud recognition index.

The latest PCA, especially, are very accurate in identifying samples of similar percentages. This confirms the fact that S3 can be used easily and quickly on the production line to make sure the 18% crust limit is not exceeded.

## 3. Experimental Section

### 3.1. S3 Device

At “SENSOR laboratory”, studies on chemical sensors begun in 1988 first with the improvement of thin films and, then, of a new technique used to plan thin films with an extremely porous structure [5]. In 2002 quasi-one-dimensional metal oxide nanostructures were achieved, thus gaining several advantages compared to their thin and thick film counterparts, such as large surface-to-volume ratio, lateral dimensions comparable to the surface charge region and superior stability when they are single crystal.

**Figure 5:**
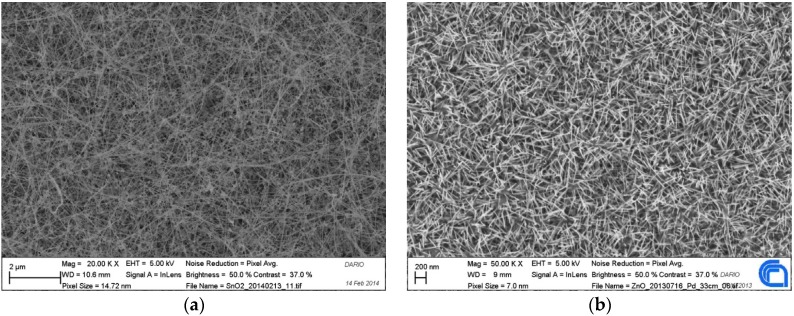
Nanowire (**a**) SnO_2_; and (**b**) ZnO SEM images.

The sensor device installed on the Small Gas Sensor System is based on an alumina transducers 2 × 2 mm^2^ with a Pt heater on the backside and thin film or NWs layer of SnO_2_ (Figure 5a) or ZnO (Figure 5b) deposited on the front side.

Thin film devices were realized by depositing a metal layer on the front side, following the Rheotaxial Growth Thermal Oxidation (RGTO) technique.

NW devices, instead, realized depositing, through the use of the PVD method (A), on the front side, exhibit remarkable crystalline quality, and a very high length-to-width ratio, resulting in enriched sensing performance, as well as long-term stability for sustained operations [13].

The Small Gas Sensor System (S3) was equipped with an array of six MOX sensors, as reported in Table 2.

**Table 2:** Preparation and working data of the electronic device sensor array (TF = Thin Film, NW = Nanowire).

Type	Sensing Layer	Catalyst	Operating Temperature (°C)
TF	SnO_2_	Au	350
TF	SnO_2_	Ag	350
NW	ZnO	–	450
NW	ZnO	–	350
TF	SnO_2_	–	450
NW	SnO_2_	–	450

S3 measurements were carried out by a dynamic headspace using an automated sampling system equipped with pure synthetic air.

### 3.2. Sampling

Two samples of pure crust, identified as CG2 and CG3, and five samples of pure pulp, identified as PG1-PG2-PG3-PG4-PG5, were analyzed. The five pulp bags and the two crusts were obtained from different forms of 12-month ripening cheese.

Five mixes of samples were analyzed: PG1-CG2, CG3-PG2, PG3-CG3, PG4-CG3, PG5-CG3.

For each mix of grated sample, the following percentages have been proposed for pulp analysis: 100% (0% crust), 95% (5% crust), 90% (10% crust), 85% (15% crust), 82% (18% crust), 81% (19% crust), 80% (20% crust), 75% (25% crust), 70% (30% crust), 65% (35% crust), 50% (50% crust), and 100% crust.

All the samples were analyzed through water activity methods. Samples have constant Aw (activity water) values and normal water activity; therefore, there is no effect on the results of the S3 device (Table 3).

**Table 3:** List of analyzed samples and correlated water activity.

Samples	Aw
PG2	0.831
PG3	0.844
PG4	0.818
PG5	0.848
CG3	0.815

Samples were manually mixed to obtain a homogeneous blend in different percentages, working in sterility and making it sure that, when ready, vials could be immediately crimped with a silicone/aluminum septum.

### 3.3. GC-MS with SPME

The mixture was then analyzed by gas chromatography (one analysis per mix) using gas chromatograph GC2010 PLUS (KYT, Kyoto, Japan) and a Shimadzu mass spectrometer coupled to a single quadrupole MS-QP2010 (KYT, Kyoto, Japan) and to the S3 device (Sensor Lab, Brescia, Italy) in parallel.

Both instruments were coupled to auto sampler HT280T (HTA srl, Brescia, Italy) to minimize mistakes in the operator’s manual preparation and heating of the sample.

Samples crimped in the vials were then incubated in an auto sampler oven at 50 °C for 15 min, in order to create the headspace equilibrium. To extract the volatile compounds of the samples, a DVB/Carboxen/PDMS stable flex SPME fiber was used. In order to provide the adsorption of volatile compounds, the SPME fiber was placed in the injector of the heater GC for 6 min at 200 °C. Volatile organic compounds were separated using an analytical capillary Colum (DB-WAX capillary column, 30 m × 0.25 mm × 0.25 µm) and the carrier gas was ultrapure helium (99.99%) at a constant flow rate of 1.5 mL/min. The temperature program for the GC was performed as it follows: from 40 °C for 8 min, followed by a linear gradient 4 °C/min to 190 °C and held for 5 min, followed by a rise from 190 °C to 210 °C at 5 °C/min and held for 5 min.

## 4. Conclusions

This work has provided very significant operational guidelines based on the application of sophisticated analytical methods, research, and determination of specific markers of recognition (everything done through SPME-GC-MS).

In addition, the development of S3 was essential: the tool, together with gas chromatography and mass spectrometry allows, to quickly understand if a sample of grated incognito is according to law [6,7]. As a matter of fact, gas chromatography/mass spectrometry allows us to identify what molecules are important in providing the flavor of Parmigiano Reggiano and, thus, training S3 to differentiate samples.

Once trained, S3 is able to recognize samples in a few minutes, without destroying them.

The olfactory fingerprint that the samples can produce can be classified starting from the comparison with “olfactory experiences” previously stored and statistically handled by the equipment [8,9,10].

The use of these devices cannot replace conventional analytical techniques but it can be integrated to perform routine checks providing, in real time, an objective judgment independent of human factors. The discrimination ability among different types of cheese in a specially calibrated electronic nose showed very satisfactory detection rates.

Different PCAs carried out for the different mix of samples, that are the result of the analysis of the S3 device, allow us to identify and distinguish the different percentages mix in a gradually clearer way as the time of calibration passes by.

The typical fraud that can affect Parmigiano Reggiano cheese is an excess in crust, as crust < 18% is a clear requirement: therefore S3 device, proved to be able to quickly recognize if rind quantity exceeds the limit.

In this paper we have shown that with a thin films/nanowires MOX EN array it is possible to distinguish the percentage of grated PR, even when very close to law limits (18% and 19%).

In our future works, we want to evaluate the percentage of grated PR in different maturation (seasoning) periods. This will be possible keeping account of the presence of VOCs released from the wood used during the seasoning period [11,12].
